# Evaluation of a commercial E^rns^-capture ELISA for detection of BVDV in routine diagnostic cattle serum samples

**DOI:** 10.1186/1751-0147-49-7

**Published:** 2007-03-13

**Authors:** Jaruwan Kampa, Karl Ståhl, Lena HM Renström, Stefan Alenius

**Affiliations:** 1 Department of Clinical Sciences, Swedish University of Agricultural Science (SLU), SE-75007, Uppsala, Sweden; 2 Faculty of Veterinary Medicine, Khon Kaen University, 40002, Thailand; 3 Department of Biomedical Sciences and Veterinary Public Health, SLU, SE-75007, Uppsala, Sweden; 4 National Veterinary Institute (SVA), SE-75007, Uppsala, Sweden

## Abstract

**Background:**

Bovine viral diarrhoea virus (BVDV) is an important pathogen in cattle. The ability of the virus to cross the placenta during early pregnancy can result in the birth of persistently infected (PI) calves. These calves shed the virus during their entire lifespan and are the key transmitters of infection. Consequently, identification (and subsequent removal) of PI animals is necessary to rapidly clear infected herds from the virus. The objective of this study was to evaluate the suitability of a commercial E^rns^-capture ELISA, in comparison to the indirect immunoperoxidase test (IPX), for routine diagnostic detection of BVDV within a control programme. In addition, the effect of passive immunity and heat-inactivation of the samples on the performance of the ELISA was studied.

**Methods:**

In the process of virus clearance within the Swedish BVDV control programme, all calves born in infected herds are tested for virus and antibodies. From such samples, sent in for routine diagnostics to SVA, we selected 220 sera collected from 32 beef herds and 29 dairy herds. All sera were tested for BVDV antigen using the E^rns ^ELISA, and the results were compared to the results from the IPX used within the routine diagnostics.

**Results:**

All 130 samples categorized as virus negative by IPX were tested negative in the ELISA, and all 90 samples categorized as virus positive were tested positive, i.e. the relative sensitivity and specificity of the ELISA was 100% in relation to IPX, and the agreement between the tests was perfect.

**Conclusion:**

We can conclude that the E^rns ^ELISA is a valid alternative that has several advantages compared to IPX. Our results clearly demonstrate that it performs well under Swedish conditions, and that its performance is comparable with the IPX test. It is highly sensitive and specific, can be used for testing of heat-inactivated samples, precolostral testing, and probably to detect PI animals at an earlier age than the IPX.

## Background

Bovine viral diarrhoea virus (BVDV) is a widely spread cattle pathogen with a significant economic impact on cattle production [1]. The virus interferes with reproductive and immunological functions and causes subsequent losses due to reproductive disorders and impaired herd performance [2,3]. Based on phylogenetic comparison, the virus can be classified into two genotypes: BVDV-1 and BVDV-2. Whereas BVDV-1 has a world-wide distribution, BVDV-2 appears to be highly prevalent only in North America [4,5] and relatively rare in other continents [6,7].

The ability of the virus to cross the placenta during the first trimester of pregnancy can result in the birth of immunotolerant and persistently infected (PI) calves. These PI calves shed the virus during their entire lifespan and are the key transmitters of virus in an infected herd [8] and responsible for maintaining BVDV infections in cattle populations [9]. Consequently, identification (and subsequent removal) of PI animals is necessary to rapidly clear infected herds from the virus.

To identify PI animals, virus isolation on primary bovine cells, followed by immuno-enzyme staining is regarded as the "gold standard" method. The indirect immunoperoxidase (IPX) test is a standard method to detect BVDV in several diagnostic laboratories and is used in the BVDV control programme in Sweden [10]. During the course of this programme, the IPX test has shown to be highly effective for identifying PI animals [11,12]. However, the IPX test is time consuming and requires a high investment both in personal training and laboratory equipment, which is why antigen-capture ELISAs have been increasingly used since the early 1990's. The NS2/3-capture ELISA detects BVDV in leukocytes and tissue samples using specific affinity monoclonal antibodies (MAb) against the NS2/3 protein, and has been successfully used to identify PI animals in BVDV control programmes in Norway and in the Shetland islands [13,14]. Recently, an antigen ELISA that uses MAbs against the E^rns ^glycoprotein has been developed to detect BVDV. This structural protein is secreted from infected cells during virus replication and can be detected directly in serum which allows user friendly and high throughput testing and gives it the potential to be a diagnostic antigen [15,16].

The objective of this study was to evaluate the suitability of a commercial E^rns^-capture ELISA (E^rns ^ELISA; HerdCheck BVDV antigen/Serum Plus, IDEXX Scandinavia AB, Österbybruk, Sweden.), in comparison to IPX, for routine diagnostic detection of BVDV within a control programme. In addition, the effect of passive immunity and heat-inactivation of the samples on the performance of the ELISA was studied.

## Methods

### 1. Selection of samples

#### 1.1. Samples from the field

In the process of virus clearance within the Swedish BVDV control programme, all calves born in infected herds are tested for virus and antibodies at an age of 12 weeks, or older. Blood samples are collected in sterile 5-ml vacutainer tubes and sent for analysis to the National Veterinary Institute (SVA), Uppsala, Sweden. From each herd detected as infected, one virus positive sample is selected for further analysis and genotyping of the infecting strain [17]. So far, only BVDV-1 has been detected in Sweden.

From samples sent in for routine diagnostics to SVA between September 2002 and February 2003, we selected 220 sera collected from 32 beef herds and 29 dairy herds throughout Sweden. According to the results from the IPX used within the routine diagnostics [18] 90 of the sera were considered virus positive and 130 virus negative. All sera were kept at -20°C until analyzed by the E^rns ^ELISA.

#### 1.2. Samples from PI calves with or without passive immunity

To study the influence of passive immunity on the performance of the ELISA and IPX, we selected serum samples from nine PI calves born after a previously described experimental infection of pregnant heifers [19]. In total, we tested 23 samples collected between day 0 (i.e. immediately after birth and before intake of colostrum) and day 11 post partum (Table 1). Of the nine calves, five (calves 1–5) were given colostrum free from BVDV antibodies, and four (calves 6–9) were given colostrum from their respective antibody positive dams. Antibody titres were determined in all sera using a commercial indirect ELISA (BVDV-Ab SVANOVIR™, SVANOVA Biotech AB, Uppsala, Sweden).

**Table 1:** Results from testing for BVDV in sera collected between days 0 and 11 post partum from nine PI calves born after experimentally infected heifers, using a commercial E^rns^-capture ELISA (HerdCheck BVDV antigen/Serum Plus, IDEXX Scandinavia AB, Österbybruk, Sweden.) and the indirect immunoperoxidase test (IPX).

PI calf no.	Age (days)	Antibody titre	Virus detection		
			IPX	E^rns^ELISA^a^	(COD)
1	0	<1:10	-	+	(3.32)
	2	<1:10	+	+	(2.96)
	7	<1:10	+	+	(2.70)
2	0	<1:10	+	+	(2.99)
	2	<1:10	+	+	(3.52)
	3	<1:10	+	+	(3.42)
3	0	<1:10	-	+	(3.21)
	2	<1:10	+	+	(3.00)
	7	<1:10	+	+	(2.97)
	11	<1:10	+	+	(2.52)
4	0	<1:10	+	+	(3.33)
5	0	<1:10	-	+	(3.56)
	2	<1:10	+	+	(3.26)
6	0	<1:10	+	+	(3.27)
	2	1:250	-	-	(0.16)
	5	1:250	-	+	(3.29)
	7	1:10	-	+	(3.28)
7	2	1:250	-	+	(0.48)
8	4	1:1250	-	-	(0.29)
	6	1:250	-	+	(1.58)
9	2	1:1250	-	+	(2.92)
	7	1:1250	-	-	(0.04)
	9	1:1250	-	-	(0.07)

^Calves 1–5 were given colostrum free from BVDV antibodies, and calves 6–9 were given colostrum from their respective antibody positive dams. Samples collected day 0 were taken before intake of colostrum. BVDV antibody titres were determined using a commercial indirect ELISA (SVANOVA Biotech AB, Uppsala, Sweden) in dilutions 1:10–1:1250.^

^a ^Samples with COD > 0.30 as measured by the Erns ELISA were considered positive

#### 1.3. Heat inactivated sera

To study the influence of heat inactivation, we selected a subset of 20 sera (10 virus positive and 10 virus negative) out of the 220 samples previously selected from the routine diagnostics. Each sample was divided in two parts, and one was heat inactivated at 56°C for 90 minutes before further analysis. Heat inactivated and non-heated sera were then tested in parallel with the E^rns ^ELISA.

### 2. Diagnostic methods

#### 2.1. Detection of BVDV by IPX

The 220 samples from the field and the 23 samples from PI calves were also tested for BVDV by IPX. The test was carried out on 96-well plates using low-passage bovine turbinate cells. Serum (20 μl) was added to each of four wells before the addition of 100 μl of cell suspension. Positive and negative control sera were run on each plate. The test plates were incubated for 4 days in 5% CO_2_, 37°C. Plates were fixed and dried, then stained with immunoperoxidase as described by Meyling [18], using a polyclonal bovine anti-BVDV serum (BVD virus positive control serum, VLA, Weybridge, UK) to detect the virus. The presence of red-brown cytoplasmic staining in any of the wells exposed to the specific anti-BVDV antibody denoted a positive result.

#### 2.2. Detection of BVDV by antigen ELISA

All samples were tested for BVDV antigen using the E^rns^-capture ELISA according to the manufacturer's instructions. Briefly, provided detection antibodies were added to all wells of a microtitre plate wells coated with E^rns ^MAbs. Positive and negative control sera were added to appropriate duplicate wells, and the serum samples (50 μl) then added to the remaining wells. The plate was incubated for 2 hours at 37°C, before washing and addition of conjugate and substrate. The optical density values (ODs) were measured at 450 nm, and the corrected optical densities (CODs) of samples and positive control then calculated by subtracting the mean OD for the negative controls from obtained OD (COD = OD^obtained ^- mean OD^negative controls^). Samples with COD> 0.30 were classified as positive.

#### 2.3. Detection of antibodies

The antibody titres of the sera from the nine PI calves were determined using the commercial indirect ELISA in dilutions 1:10 to 1:1250. The ELISA was performed according to the instructions of the manufacturer. The COD values from the indirect ELISA (COD^Ab^) were calculated before interpretation of the result by subtracting the OD for control antigen from obtained OD (COD^Ab ^= OD^obtained ^- OD^control^). Antibody titres were determined as the highest dilution with COD^Ab ^≥ 0.2.

### 3. Statistical analysis

The sensitivity (Se) and specificity (Sp) of the E^rns ^ELISA were calculated in relation to the IPX test, and the degree of agreement between the tests was estimated by the kappa (*k*) ratio. To estimate the effect of heat inactivation on the performance of the ELISA we evaluated the repeatability of the test before and after heat inactivation of the samples using the concordance correlation coefficient (CCC) [20] and Bland-Altman plot [21].

## Results

### Samples from the field

**Figure 1:**
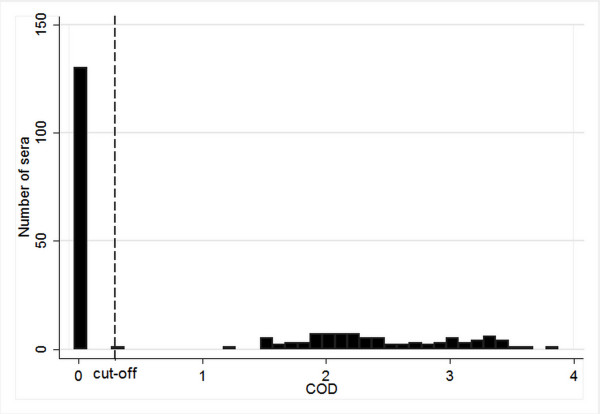
Frequency distribution diagram of corrected optical density (COD) values as measured by a commercial E^rns^-capture ELISA (HerdCheck BVDV antigen/Serum Plus, IDEXX Scandinavia AB, Österbybruk, Sweden) in 90 bovine sera considered as virus positive and 130 bovine sera considered as virus negative according to the indirect immunoperoxidase test used within the Swedish BVDV control programme. All sera were selected among samples sent for routine diagnostics to the National Veterinary Institute, Uppsala, Sweden, between September 2002 and February 2003. ELISA results with COD > 0.30 were considered positive.

The results from the testing of the 220 sera, selected among samples sent in to SVA, are summarized in Table 2. All 130 samples categorized as virus negative by IPX were tested negative in the ELISA, and all 90 samples categorized as virus positive were tested positive, i.e. the relative Se and Sp of the ELISA was 100% in relation to IPX, and the agreement between the tests was perfect (*k *= 1.0). The frequency distribution of COD values can be seen in Figure 1. Out of 90 samples considered as virus positive according to the IPX test results, 89 had COD values well above the cut-off (>1.2).

**Table 2:** Results from testing of 220 Swedish bovine serum samples using a commercial E^rns^-capture ELISA (HerdCheck BVDV antigen/Serum Plus, IDEXX Scandinavia AB, Österbybruk, Sweden.), and comparison with results obtained with the indirect immunoperoxidase (IPX) test.

E^rns ^ELISA		IPX	
	Mean COD (range)	Negative	Positive
Negative	0.00 (-0.027 – 0.03)	130	0
Positive^a^	2.34 (0.31–3.78)	0	90

^a ^ELISA results with COD > 0.30 were considered positive

### Samples from PI calves with or without passive immunity

The results of the testing of the 23 samples from PI calves are given in Table 1. Out of 14 samples with antibody ELISA titres < 1:10, 14 were tested positive in the E^rns ^ELISA and 11 in the IPX. The three samples that were falsely classified as negative by the IPX were all collected day 0 post partum, i.e. before intake of colostrum. Among the 9 samples with antibody titres ranging between 1:10 and 1:1250, 5 samples were tested positive in the E^rns ^ELISA and none in the IPX.

### Heat inactivation

**Figure 2:**
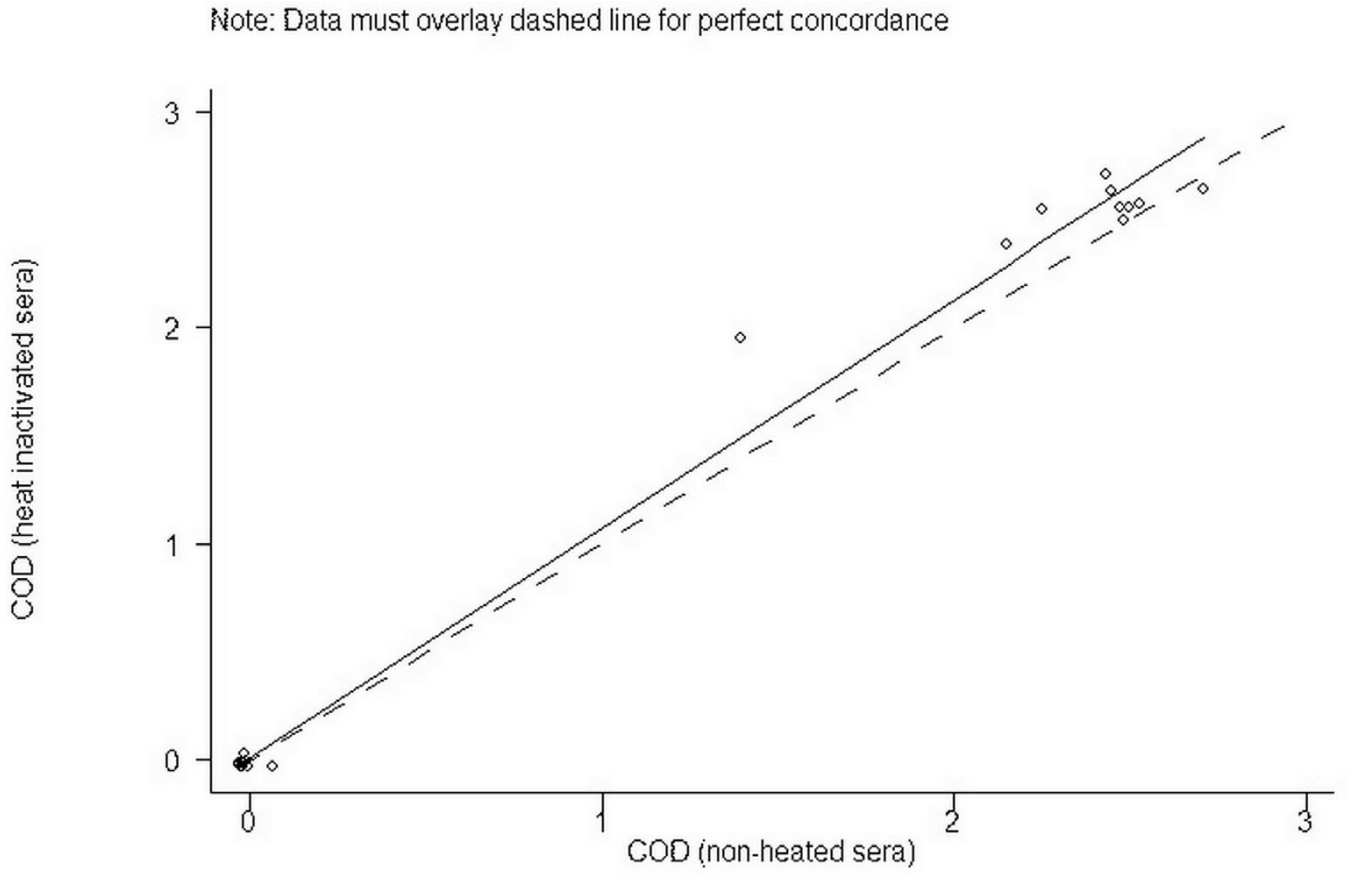
Agreement between COD values obtained with a commercial E^rns^-capture ELISA (HerdCheck BVDV antigen/Serum Plus, IDEXX Scandinavia AB, Österbybruk, Sweden.) from testing of 20 bovine serum samples (10 virus positive and 10 virus negative) before and after heat inactivation at 56°C for 90 minutes (CCC = 0.99).

The agreement between results obtained before and after heat inactivation can be seen in Figure 2. There was a perfect agreement of the interpretations of the test results obtained before and after heat inactivation, and the CCC was calculated to 0.99. The BA plot (not shown) indicated that 95% of the differences between heat-inactivated and non-heated samples fell in the range of -0.39 and +0.22 units.

## Discussion

The identification of PI animals (for subsequent elimination) is an essential element in any BVDV control programme, and depends on accurate diagnostic tests, i.e. tests with high sensitivity and specificity that have been thoroughly evaluated for routine diagnostic use. Moreover, for testing of large series of samples it is desirable that a test is user friendly and allows automation. Even though the IPX test currently used in Sweden has shown to be efficient for detection of PI animals, it is evident that the E^rns ^ELISA have several advantages: it is independent of cell cultures, gives a test result within a few hours and is relatively inexpensive both to establish and run [22]. In addition, our results clearly demonstrate that it performs well under Swedish conditions, i.e. for detection of BVDV-1, and that its performance is comparable with the IPX test. There was a perfect agreement between the results from the two tests, and the separation between COD values from negative and positive samples was good. Out of 220 samples, 219 had COD values either well below or well above the cut-off. However, one sample, considered as virus positive according to the IPX test results, had a COD value close to the cut-off, and there are a number of possible explanations for this result. Firstly, as with the majority of BVDV antigen ELISAs, this E^rns ^ELISA has been developed for the identification of PI animals. Whereas virus titres in PI animals normally range between 10^2.2 ^and 10^6 ^TCID_50_/ml, titres during transient infections have been reported to be as low as 10^0.9 ^[23-25]. It is likely that the detection level of the IPX test is lower than that of the ELISA, and it is possible that this serum sample originated from a transiently infected animal. Secondly, although PI animals normally have high virus titres, these may, as previously mentioned, show a wide range. Consequently, it is also possible that this serum sample originated from a PI animal, but that the virus titer was low, and close to the detection limit of the ELISA.

Because the E^rns ^ELISA, unlike the IPX test used, is based on MAbs and has been developed and validated for detection of BVDV-1 and BVDV-2, it is probably not as broadly reactive as the IPX. It has been shown not to detect some closely related border disease virus strains [26], and may, consequently, also miss atypical pestiviruses. This should be kept in mind, as there are indications that atypical pestiviruses are already circulating in cattle populations[27,28].

It was demonstrated that both tests might fail to detect a large proportion of PI calves in the presence of persisting maternal antibodies, confirming results from previous studies[29,30]. However, whereas the IPX test gave false negative results also in the presence of low antibody titres, the E^rns ^ELISA detected BVDV in three out of four sera with antibody titres up to 1:250, indicating that the E^rns ^ELISA is less influenced by passive immunity. We could also observe that the IPX test, unlike the E^rns ^ELISA, gave false negative results in three out of five newborn PI calves sampled before intake of colostrum. This has been observed previously and is hard to explain (Rønsholt, personal communication), but is one of several reasons for which precolostral sampling is not practiced within the Swedish BVDV control programme.

In addition, it was demonstrated that the performance of the E^rns ^ELISA was not influenced by heat inactivation, which can be an advantage in laboratories where sera are often subject to several analyses and therefore heat-inactivated by routine.

## Conclusion

Based on these results we can conclude that the E^rns ^ELISA is a valid alternative to the IPX test. It is highly sensitive and specific, can be used for testing of heat-inactivated samples, precolostral testing, and probably to detect PI animals at an earlier age than the IPX. However, it should be kept in mind that this ELISA, unlike the IPX, uses MAbs, and that it therefore is less likely to detect atypical pestivirus strains.

## Competing interests

The author(s) declare that they have no competing interests.

## Authors' contributions

JK and SA took part in all aspects of the study, including study design, laboratory analysis, interpretation of the results, and drafting of the manuscript. KS participated in interpretation of the results and drafting of the manuscript. LR participated in study design and revision of the manuscript. All authors have read and given final approval of the version to be published.
